# Mighty Mums – a lifestyle intervention at primary care level reduces gestational weight gain in women with obesity

**DOI:** 10.1186/s40608-018-0194-4

**Published:** 2018-06-04

**Authors:** Karin Haby, Marie Berg, Hanna Gyllensten, Ragnar Hanas, Åsa Premberg

**Affiliations:** 1 Primary Health Care, Research and Development Unit, Närhälsan, Region Västra Götaland, Gothenburg, Sweden; 2 Institute of Health and Care Sciences, Sahlgrenska Academy, University of Gothenburg, Gothenburg, Sweden; 3 GPCC – University of Gothenburg Centre for Person-centred Care, Gothenburg, Sweden; 4 Department of Pediatrics, NU Hospital Group, Region Västra Götaland, Uddevalla, Sweden; 5 Institute of Clinical Sciences, Sahlgrenska Academy, University of Gothenburg, Gothenburg, Sweden

**Keywords:** Pregnancy, Obesity, Lifestyle intervention, Gestational weight gain

## Abstract

**Background:**

Obesity (BMI ≥30) during pregnancy is becoming an increasing public health issue and is associated with adverse maternal and perinatal outcomes. Excessive gestational weight gain (GWG) further increases the risks of adverse outcomes. However, lifestyle intervention can help pregnant women with obesity to limit their GWG. This study evaluated whether an antenatal lifestyle intervention programme for pregnant women with obesity, with emphasis on nutrition and physical activity, could influence GWG and maternal and perinatal outcomes.

**Methods:**

The intervention was performed in a city in Sweden 2011–2013. The study population was women with BMI ≥30 in early pregnancy who received standard antenatal care and were followed until postpartum check-up. The intervention group (*n* = 459) was provided with additional support for a healthier lifestyle, including motivational talks with the midwife, food advice, prescriptions of physical activity, walking poles, pedometers, and dietician consultation. The control group was recruited from the same (*n* = 105) and from a nearby antenatal organisation (*n* = 790).

**Results:**

In the per-protocol population, the intervention group had significantly lower GWG compared with the control group (8.9 ± 6.0 kg vs 11.2 ± 6.9 kg; *p* = 0.031). The women managed to achieve GWG < 7 kg to a greater extent (37.1% vs. 23.0%; *p* = 0.036) and also had a significantly lower weight retention at the postpartum check-up (− 0.3 ± 6.0 kg vs. 1.6 ± 6.5 kg; *p* = 0.019) compared to the first visit. The most commonly used components of the intervention, apart from the extra midwife time, were support from the dietician and retrieval of pedometers. Overall compliance with study procedures, actual numbers of visits with logbook activity, and dietician contact correlated significantly with GWG. There was no statistically significant difference in GWG (10.3 ± 6.1 kg vs. 11.2 ± 6.9 kg) between the intervention and control groups in the intention-to-treat population.

**Conclusion:**

Pregnant women with obesity who follow a lifestyle intervention programme in primary health care can limit their weight gain during pregnancy and show less weight retention after pregnancy. This modest intervention can easily be implemented in a primary care setting.

**Trial registration:**

The study has been registered at ClinicalTrials.gov, Identifier: NCT03147079. May 10 2017, retrospectively registered.

## Background

In line with rising global figures for the general population, obesity in relation to pregnancy is becoming an increasing global public health issue. Across Europe, the majority of countries in 2013 had high rates of overweight and obesity in early pregnancy; Scotland showed the highest prevalence (48%) and Slovenia the lowest (18%), with Sweden in between (38%) [1].

Of women assigned to antenatal care in Sweden in 2016, 26.6% had overweight (body mass index [BMI] ≥25) and 14.1% had obesity (BMI ≥30). The prevalence was higher in pregnant women with elementary education (vs. high school or university) and women born in foreign countries [2]. Women with lower education also had the largest BMI increase between pregnancies [3].

Living in communities with low socioeconomic standards is associated with higher BMI. Moreover, women in disadvantaged neighbourhoods are more likely to gain unhealthy weight, which supports the need for improved preconception and antenatal care [4]. The well-being of the next generation is at risk, since maternal obesity is a significant factor leading to obesity in offspring, with further negative health consequences [5, 6]. Thus, even if healthy living habits are the responsibility of the individual, potential social and environmental factors involved must also be considered, so that children, youth, and women have the possibility of living healthy lives to prevent obesity and its negative consequences [4].

According to a systematic review of 22 reviews, obesity in pregnancy was associated with increased risk of gestational diabetes, preeclampsia, gestational hypertension, depression, preterm birth, large-for-gestational-age babies, congenital anomalies, instrumental and caesarean birth, perinatal death, and surgical site infection [7]. Obesity in early pregnancy was a predictor for excessive gestational weight gain (GWG) [8] and excessive GWG per se was a predictor for postpartum weight retention [8–10]. Excessive GWG has been associated with high foetal birthweight [11] and with offspring becoming overweight or obese in childhood and adolescence [12–14]. In addition, women with excessive GWG were more likely to experience postpartum weight retention and long-term obesity [8, 15], in particular, those with first-trimester weight gain [16].

To minimise the risks of negative health consequences of both inadequate and excessive GWG, American guidelines on limiting GWG have been developed by the Institute of Medicine (IOM) [17], which are used internationally. However, these guidelines have not been systematically implemented in Sweden, since a Swedish study showed that if GWG is even lower than the IOM recommendation, the increased risk of complications for both woman and offspring can be reduced, especially among women with obesity [18, 19]. The study, with almost 300,000 pregnancies, showed that a GWG below 6 kg in obese women was associated with a lower risk of adverse maternal and neonatal outcomes [18].

Programmes are being introduced in antenatal care that address obesity to prevent excessive GWG, and there has been a tendency towards decreasing GWG in Swedish women with high BMI [2]. Diet, exercise, or both can reduce the risk of excessive GWG [20], and diet- and physical activity-based interventions during pregnancy reduce GWG and lower the odds of caesarean section [21]. On one hand, evidence suggests that exercise is a strong part of controlling GWG [20], while other studies support interventions based on diet appearing to be most effective [22]. Behavioural interventions may be effective in reducing GWG in obese women during pregnancy, but the variation in interventions that have been tested makes comparisons difficult [23]. Evaluations of interventions have yielded mixed results, and specific characteristics of effective interventions are under-reported in the literature [24]. Also, there is a demand for interventions that facilitate positive future outcomes and decreased negative effects for the offspring [25]. Routine weighing alone appears not to be effective in reducing GWG, especially in women with obesity [26, 27], and there is thus a demand for implementation of evidence-based strategies to enhance healthy lifestyle in routine antenatal care [10].

The primary aim of this study was to evaluate whether a structured antenatal lifestyle intervention at primary care level for pregnant women with obesity can result in lower mean GWG; a larger proportion of women with a GWG less than the target of 7 kg, a limit used in earlier research [28]; and lower weight at the postnatal check-up, compared with women receiving standard care. The secondary aims were to study whether the intervention had impact on maternal and child perinatal health outcomes, and to identify which subcomponents of the intervention were favoured by the participants who were successful in limiting GWG.

## Methods

The Mighty Mums (MM) project was a standardised programme delivered during regular antenatal care, aiming to reduce GWG in pregnant women with obesity. Results from a pilot study have been described elsewhere [29]. Theories of empowerment [30], motivational interviewing (MI) [31], and person-centred care [32] inspired the individualised approach used in the intervention.

### Study population

The study, conducted in a city area in western Sweden over 3 years (2011–2013), involved 3300 pregnant women with BMI ≥30 at the first visit to the antenatal care. Based on the organisation of the antenatal care, the intervention was conducted in the major part of the city with 2500 pregnant women having BMI ≥30. A smaller catchment area within the city with 800 pregnant women having BMI ≥30 was assigned as a control area. After informed consent, women enrolled in the intervention group (*n* = 459) and the control group (*n* = 105) were followed from the first trimester of the pregnancy until postpartum check-up, in registers and during antenatal care.

**Fig. 1:**
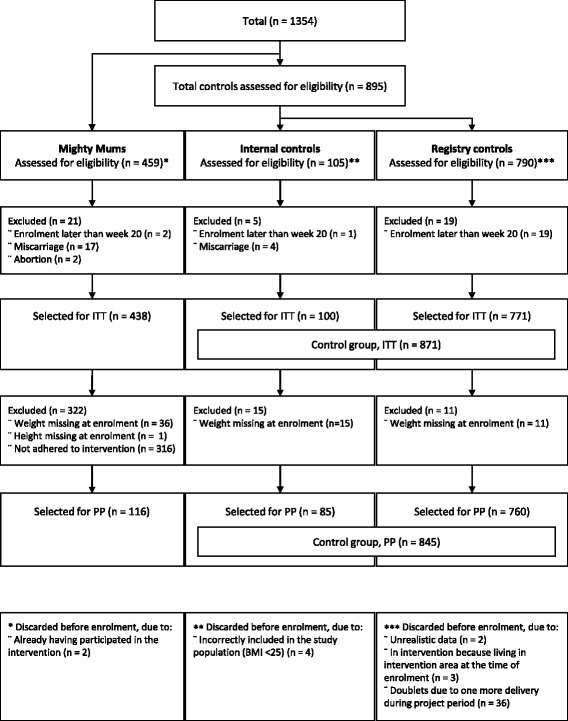
Flow chart of women in the study. ITT = intention-to-treat population; PP = per-protocol population. There is some overlap between reasons for exclusion from the PP population in the intervention group

An adjacent area with 790 pregnant women with BMI ≥30 was added to the control group. Altogether, 1354 women were enrolled, 459 in the intervention and 895 in the total control group (Fig. 1). Due to clinical routines and the medical record system, BMI was rounded off, and some women having a true BMI of less than 30 were included (*n* = 37, see Table 1).

**Table 1:** Baseline characteristics of participants

Variable	Intention-to-treat population		Per-protocol population	
	Intervention Mean (SD) Median (range) (*n* = 438)	Controls Mean (SD) Median (range) (*n* = 871)	Intervention Mean (SD) Median (range) (*n* = 116)	Control Mean (SD) Median (range) (*n* = 845)
Weeks pregnant at first pregnancy visit	8.6 (2.5) 8.2 (3–20)	7.9 (2.3) 7.9 (5–18)	8.3 (2.1) 7.9 (4–15)	7.9 (2.3) 7.7 (5–18)
Age, years	30.9 (5.5) 30.5 (18.2–47.4)	30.7 (5.1) 30.4 (17.6–46.1)	30.7 (5.4)30.1 (20.7–47.4)	30.7 (5.1) 30.3 (17.6–46.1)
Weight at first pregnancy visit, transformed to week 15, kg	94.0 (13.9) 92.0 (63.0–152.0)	93.4 (11.5) 92.0 (69.0–153.0)	94.1 (14.7) 91.0 (67.0–152.0)	93.3 (11.3) 92.0 (69.0–144.0)
Height at first pregnancy visit, cm	165.8 (7.5) 165.0 (133.0–187.0) *n* = 437	166.4 (6.2) 166.0 (148.0–185.0)	165.8 (7.2) 165.0 (148.0–180.0)	166.4 (6.2) 166.0 (148.0–185.0)
BMI at first pregnancy visit, transformed to week 15	34.1 (4.0) 33.3 (27.7–57.2) *n* = 437	33.7 (3.2) 32.8 (29.7–50.0)	34.1 (3.7) 33.1 (29.3–49.6)	33.6 (3.1) 32.8 (29.7–47.0)
	n (%)	n (%)	n (%)	n (%)
Overweight BMI < 30.0^a^	28 (6.4)	5 (0.6)	6 (5.2)	5 (0.6)
Obese Class I BMI 30.0–34.9	271 (62.0)	611 (70.1)	74 (63.8)	596 (70.5)
Obese Class II BMI 35.0–39.9	98 (22.4)	210 (24.1)	25 (21.6)	204 (24.1)
Obese Class III BMI ≥40	40 (9.2)	45 (5.2)	11 (9.5)	40 (4.7)
Primipara	204 (46.6)	338 (38.8)	63 (54.3)	326 (38.6)
Born outside Sweden	131 (29.9)	92 (10.6)	35 (30.2)	89 (10.5)
Use of translator	46 (10.5)	17 (2.0)	14 (12.1)	17 (2.0)
Education ≤12 years^b^	269 (61.6)	564 (64.8)	68 (58.6)	545 (64.6)
Other than employed^c^	151 (34.5)	194 (22.3)	43 (37.1)	187 (22.2)
Use of nicotine	33 (7.5)	79 (11.0)	8 (6.9)	77 (11.1)

Values represent mean (SD) and median (range) for continuous variables, and n (%) for categorical variables

^a^Due to clinical routines, BMI has been rounded off and some women having a true BMI less than 30 have been included, *n* = 37

^b^Below university studies

^c^Being subsidised by parental leave, unemployment benefits, student loans, or social security

### Standard antenatal care and the intervention

All women received standard antenatal care. This comprised care by a midwife during pregnancy and the postpartum visit, usually a total of nine visits to the midwife. All women’s weights were checked at the first visit, at weeks 25 and 37, and at the postnatal check-up, according to the regular antenatal programme. This also included referral to the anaesthetic unit for women with BMI ≥40 for assessment and planning of the upcoming labour and birth.

The MM project was designed to function in everyday practice and one of the fundaments was MI [31]. Women in the intervention group received additional care in the form of motivational talks and personalised counselling on food and physical activity, delivered by the midwife at two extra appointments, around 30 min each, during early pregnancy. Based on each participant’s choice, the women were also offered individualised dietary advice from a dietician, food discussion groups with a dietician, aqua aerobics led by a physiotherapist and a midwife, prescriptions for physical activity, walking poles, pedometers, and information about community health centres offering lifestyle education and lighter exercise. Apart from the two extra appointments in early pregnancy, about 5 min of each appointment with the midwife were dedicated to the follow-up of lifestyle. The woman’s weight was checked at every appointment, approximately 11 check-ups in total, including postpartum check-up.

Moreover, at one of the first visits to the midwife, food and activity habits were mapped, and a logbook was introduced. The woman and the midwife used the logbook throughout the pregnancy and at the postpartum check-up to register weight and record comments on successes and drawbacks as well as enablers and obstacles in managing the planned lifestyle changes. With the logbook it was possible for the woman and the midwife to work together in partnership with the lifestyle changes, and for the woman to take responsibility for her choices and adapt the plan to her own capacity. The activities in the programme were built on the idea that the woman should be active and take part in all decisions of the programme, which is crucial and a cornerstone in person-centred care [32].

Before the start of the project, the midwives were given education about obesity, and about current recommendations on nutrition and physical activity during pregnancy. They were also trained in MI [31] and how to use the logbook. Information on the project and advice on food and physical activity were available on the antenatal care website for the midwife to use for self-education, and to hand out to women in the intervention. A network with the surrounding community was formed, and healthcare providers and doulas (coaches for the woman during pregnancy and labour) were contacted to find areas for interaction and support. Collaboration was initiated with community health centres.

### Data collection

Data were collected from the antenatal medical records and included country of birth, language, need for interpreter, educational level, employment status, smoking status, height, weight (as measured in light clothing on a digital scale in the antenatal clinic), mode of delivery and the child’s weight and Apgar score (numerical summary of the health of the newborn). Information on pregnancy complications (gestational hypertension, preeclampsia, gestational diabetes) was gathered from the antenatal record. Data on the intervention were collected from the logbook. The weight measured at the first antenatal visit was used to calculate baseline BMI. The information on education was collected from the national maternity health register.

Weight at the first visit to antenatal care was transformed to week 15 using data from the national maternity health register, if first weight was measured after week 15 (*n* = 11) [33]. For missing data on postpartum weight, stochastic imputation was performed using fully conditional specifications (FCS) with seed = 4918. GWG was calculated as the difference between weight at the postpartum check-up and first visit weight.

### Analyses

The main analyses were comparisons between the total intervention and control groups (intention to treat analyses, ITT), including all women and adjusted for significant confounders (*p* ≤ 0.05), including weeks pregnant at first visit, height, country of birth (mother), need of translator, main occupation, and BMI at first visit transformed to 15 weeks of pregnancy. The adjusted mean differences, for GWG and secondary outcome variables, were estimated with 95% confidence intervals. Analyses included multivariable binary logistic regression for dichotomous variables, analysis of covariance (ANCOVA) for normally distributed continuous variables, and multivariable binary logistic regression for non-normally distributed continuous variables and ordered categorical variables, respectively. Correlations for adherence to the intervention were performed using Spearman’s correlation coefficient.

To address potential lack of adherence to the programme, and to the standard antenatal care, additional analyses were conducted for an identified per-protocol (PP) population. Women were included in the PP population if they had registered weight and height at first visit to antenatal care and registered last weight in pregnancy. For the women in the intervention, it was furthermore required that they had participated at a defined minimum level: adherence to activities with food and physical activity, with at least level 2 (of 1–4 where 1 is “not followed” and 4 is “followed”), according to at least three (of six possible) notifications in the logbook. The criteria for the intervention group were established before statistical analyses were performed. A composite variable was constructed, indicating the number of activities that each woman chose to participate in.

### Power calculation

With 100 women in each group, the power of this study was 80% for finding a difference between groups of at least 1.1 kg at a significance level of 0.05.

## Results

### Characteristics of the study participants

Descriptive data for the women’s baseline characteristics are given in Table 1. Significant differences were seen between the intervention group and controls, for the ITT population with regard to country of birth, need of translator, employment status, and BMI at enrolment, and for the PP population, to country of birth, use of translator, and employment. These variables were controlled for in the statistical analyses.

### Gestational weight gain

**Fig. 2:**
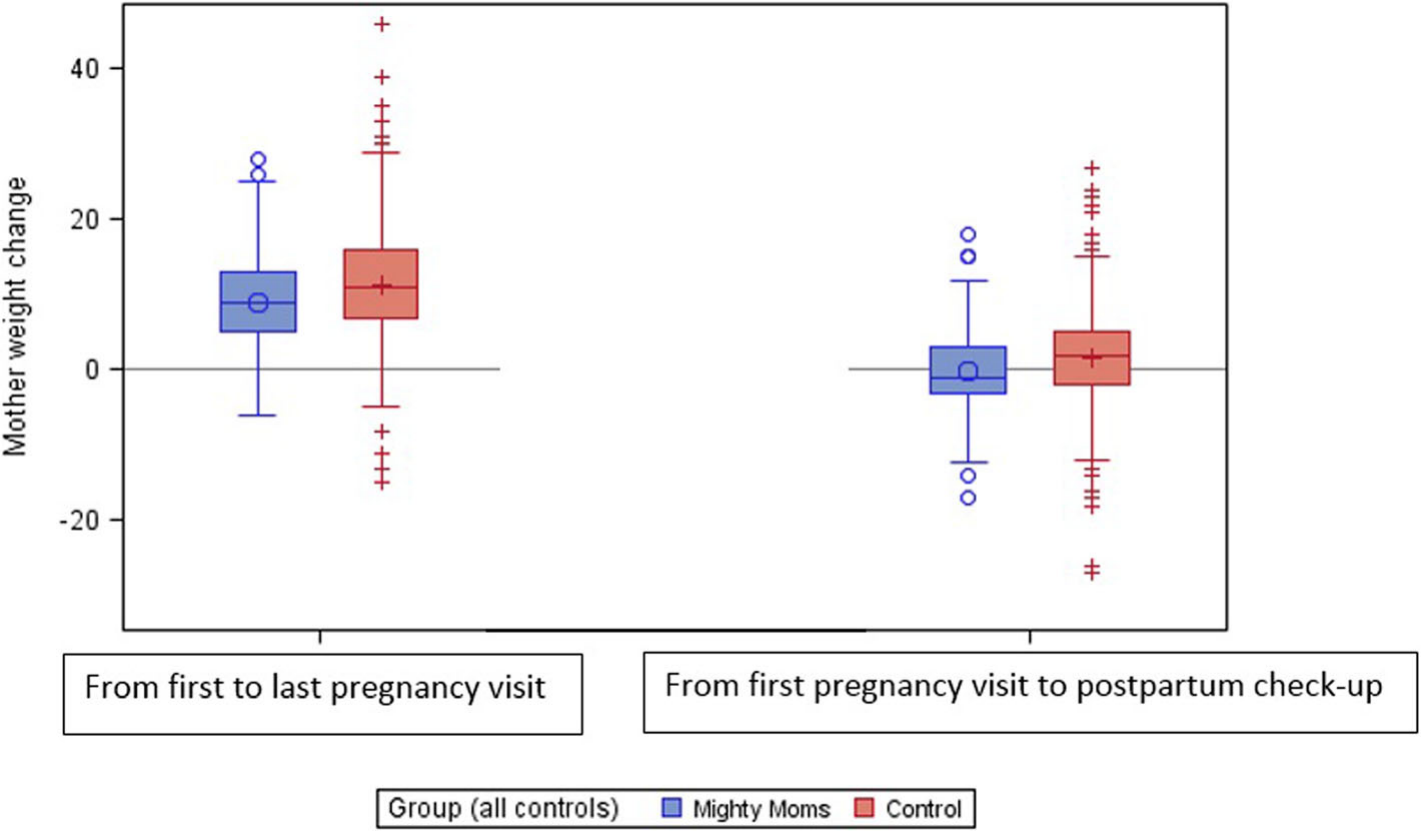
Change in mothers’ weight during and after pregnancy, by group (PP)

**Fig. 3:**
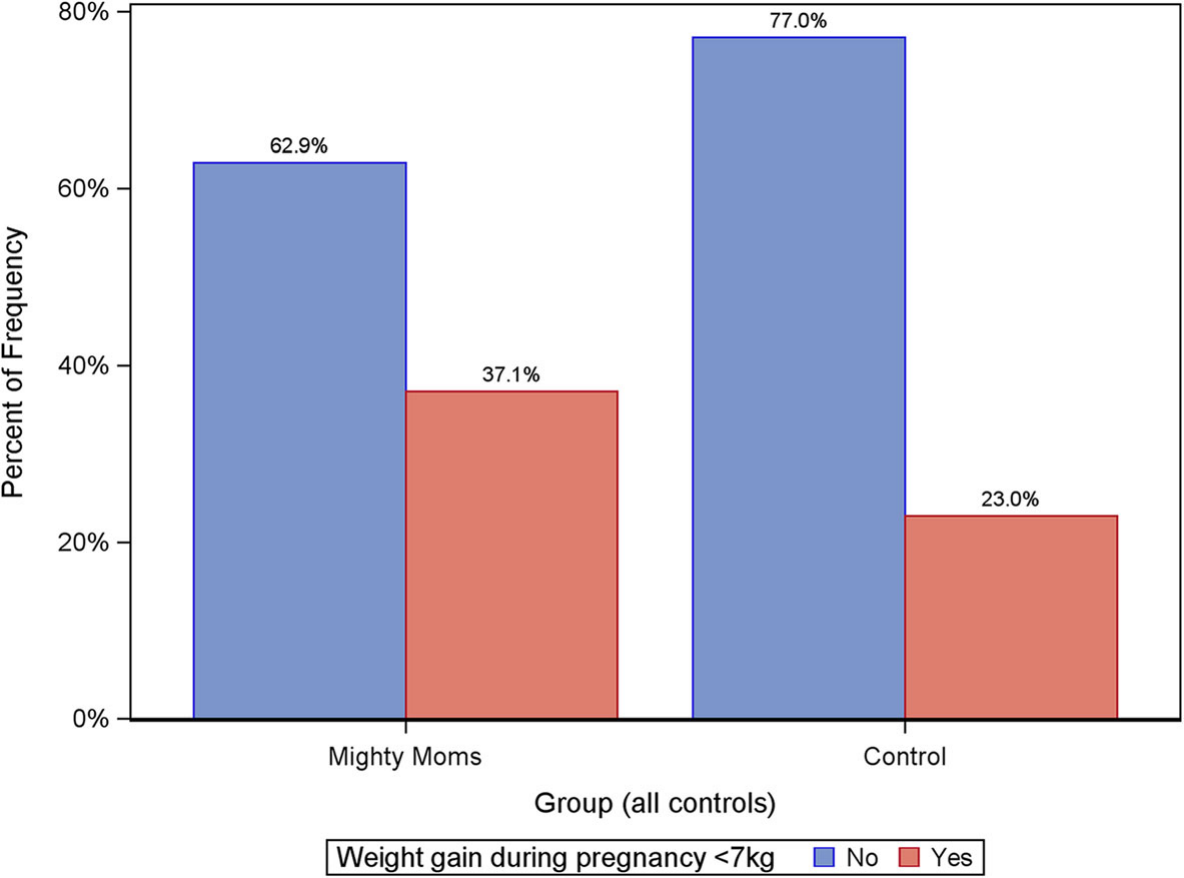
Gestational weight gain < 7 kg, by group (PP)

The PP analysis (Table 2) showed that the women in the intervention group had a significantly lower GWG compared to controls (8.9 ± 6.0 kg vs 11.2 ± 6.9 kg; *p* = 0.031) (Fig. 2). A significantly larger number of these women managed GWG < 7 kg (37.1% vs. 23.0%; *p* = 0.036) (Fig. 3), and also had a significantly lower weight retention at postpartum check-up (− 0.3 ± 6.0 kg vs. 1.6 ± 6.5 kg; *p* = 0.019) (Fig. 2). There were no significant differences for variables connected to birth size in the PP population.

**Table 2:** Results from the per-protocol and intention-to-treat analyses

Variable	Intention-to-treat population			Per-protocol population		
	Intervention Mean (SD) Median (range) (*n* = 438)	Controls Mean (SD) Median (range) (*n* = 871)	Adjusted *p*-value^a^	Intervention Mean (SD) Median (range) (*n* = 116)	Controls Mean (SD) Median (range) (*n* = 845)	Adjusted *p*-value^a^
Week of delivery	39.1 (2.5) 40.0 (24–42) *n* = 429	39.8 (2.0) 40.0 (23–42) *n* = 866	0.001	39.6 (1.5) 40.0 (36–42)	39.8 (2.0) 40.0 (23–42)	0.142
Weight change: from first pregnancy visit to last pregnancy visit, kg	10.3 (6.1) 10.0 (−6.0–41.0)	11.2 (6.9) 11.0 (−15.0–46.0)	0.695	8.9 (6.0) 9.00 (−6.0–28.0)	11.2 (6.9) 11.0 (−15.0–46.0)	0.031
Weight change: from first pregnancy visit to postpartum check-up, kg	1.4 (6.4) 1.0 (−19.0–23.0)	1.6 (6.5) 2.0 (−27.0–27.0)	0.731	−0.3 (6.0) −1.0 (− 17.0–18.0)	1.6 (6.5) 2.00 (−27.0–27.0)	0.019
Child weight at delivery, g	3591 (594) 3605 (830–5430) *n* = 420	3695 (637) 3705 (418–5760) *n* = 866	0.037	3603 (505) 3515 (2480–5430) *n* = 113	3703 (627) 3705 (418–5760)	0.300
	n (%)	n (%)		n (%)	n (%)	
GWG < 7 kg	120 (27.4)	204 (23.4)	0.882	43 (37.1)	194 (23.0)	0.036
Macrosomia	22 (5.0)	77 (8.8)	0.017	5 (4.3)	76 (9.0)	0.172
SGA^b^	34 (7.8)	45 (5.2)	0.196	10 (8.6)	38 (4.5)	0.199

Values represent mean (SD) and median (range) for continuous variables, and n (%) for categorical variables

^a^Adjusted for weeks pregnant at enrolment, height at enrolment, country of birth (mother), translator needed, main occupation, and BMI at enrolment transformed to 15 weeks

^b^Small for gestational age

In the ITT population (Table 2) there was a slightly, but not significantly, lower GWG compared to the control group (10.3 ± 6.1 kg vs. 11.2 ± 6.9 kg) and 27.4% of women in the intervention group managed to keep GWG < 7 kg in comparison with 23.4% among controls. Child weight was significantly higher, and macrosomia (i.e. birth weight > 4500 g) significantly more common in the control group.

Overall, the prevalence of adverse maternal outcomes (gestational diabetes, gestational hypertension, and preeclampsia) and perinatal outcomes (preterm delivery, intrauterine foetal death, caesarean delivery, Apgar) did not differ significantly between groups.

### Adherence to the programme

Maximum attendance (Table 3) implied seven notifications in the logbook, corresponding to seven discussions on the topic with the midwife: one initial visit, five follow-ups throughout the pregnancy, and one at the postpartum check-up. Of the women in the intervention (*n* = 438), 27.2% (*n* = 119) fulfilled the criterion of adherence to the study protocol, that is, fulfilled the prescribed activities at level two on at least three follow-ups with the midwife during pregnancy. All extra activities were optional; 39.0% (*n* = 170) had contact with the dietician (individually or in food discussion groups), 34.7% (*n* = 148) used pedometers, 20.0% (*n* = 86) used walking poles and 16.9% (*n* = 73) participated in aqua aerobics. Most women chose to organise physical activities on their own, and the most common activity was walking, often on a level of 30 min 5–7 days a week. The mean number of visits with logbook activity was higher (6.3 ± 0.6) in the PP population than in the ITT population (4.7 ± 2.3). Dietician counselling and use of walking poles and pedometers as well as participation in aqua aerobics were more common in the PP population, and this group also had a slightly higher score concerning the composite variable for all activities (4.3 ± 1.1 vs. 3.5 ± 1.7).

**Table 3:** Adherence to the Mighty Mums study protocol

Variable	Intention-to-treat population	Per-protocol population
	Mean (SD) Median (range) *n* = 438	Mean (SD) Median (range) *n* = 116
Food adherence^a^, of all visits	2.9 (0.8) 3 (1–4) *n* = 346	3.2 (0.7) 3 (2–4)
Physical activity adherence^a^, of all visits	2.5 (0.8) (1–4) *n* = 356	2.8 (0.6) 3 (2–4)
Number of logbook visits	4.7 (2.3) 6 (0–7)	6.3 (0.6) 6 (5–7)
Composite variable for all activities	3.5 (1.7) 4 (0–7)	4.3 (1.1) 4 (3–7)
	n (%)	n (%)
Adherence^a^ to both food and physical activity criteria	119 (27.2)	116 (100)
Adherence^a^ to food criteria	276 (63.0)	116 (100)
Adherence^a^ to physical activity criteria	295 (67.4)	116 (100.0)
Use of pedometer	148 (34.7)	45 (38.8)
Use of walking poles	86 (20.0)	34 (29.3)
Contact with dietician	170 (39.0)	49 (42.2)
Participated in aqua aerobics	73 (16.9)	24 (20.7)
At least one visit with follow-up of food activities	333 (76.0)	116 (100)
At least one visit with follow-up of physical activity	317 (72.4)	116 (100)
At least one logbook visit	391 (89.3)	116 (100)
Number of logbook visits		
0–4	136 (30.9)	0 (0)
5–6	220 (50.2)	70 (60.3)
7	82 (18.7)	46 (39.7)

Values represent mean (SD) and median (range) for continuous variables, and n (%) for categorical variables

^a^Adherence = at least level 2 on at least three visits according to registration in logbook

Overall compliance with study procedures (number of visits with both food and physical activity on at least level 2) correlated significantly with GWG (Table 4), as did actual numbers of visits with logbook activity and having contact with the dietician. Participating in activities with physical activity (i.e. pedometers, walking poles, and aqua aerobics) did not correlate with GWG.

**Table 4:** Correlation between adherence and weight gain among women in the intervention group, ITT population

Variable	Number of observations	Spearman correlation coefficient	*P*-value
Adherence^a^ to both food and physical activity criteria	402	−0.157	0.002
Number of visits with adherence^a^ to both food and physical activity criteria	402	−0.162	0.001
Adherence^a^ to food criteria	402	−0.127	0.011
Number of visits with adherence^a^ to food criteria	402	−0.129	0.010
Adherence^a^ to physical activity criteria	402	−0.119	0.017
Number of visits with adherence^a^ to physical activity criteria	402	−0.179	< 0.001
Contact with dietician	400	−0.122	0.015
Number of logbook visits	402	−0.169	0.001

^a^Adherence = above level 1 on more than two visits according to registration in logbook

The logbook gave an idea of which food advice was agreed upon and how it was discussed. Most midwives gave general food advice from the website, but it was also common to note individual advice in the logbook: “restrict carbohydrates”, “eat regularly”, “cut out sweets and sweet drinks”, and more positively, “increase fruit and vegetables”, “eat fish”, and “savour the food”.

## Discussion

This study shows that an antenatal care programme resulted in a significantly lower GWG, significantly lower weight retention at the postnatal check-up, and significantly more women being successful in limiting GWG to less than 7 kg if they followed the individually planned lifestyle changes.

The results from this study are in line with other lifestyle studies where effect on GWG has been shown after nutritional advice alone, or in combination with advice on physical activity [28, 34–37]. Interesting findings from trials seem to be that the effect of getting information from brochures, seminars, and websites should not be underestimated [35, 37, 38], and that more intense interventions do not always give the best results [28, 36]. One explanation may be that delivery of objective information in group settings or electronically is successful, since pregnant women with BMI ≥30 have the experience of being addressed in a judgemental way about their weight, and request accurate and appropriate information about the benefits of limited gestational weight gain [39].

Several reviews conclude that behavioural GWG interventions, even if successful, should be more systematically designed and evaluated, as well as based on insights from behavioural science [22, 24, 40, 41]. The MM project was designed to function in structured everyday practice, and one of the fundaments was the skill in MI that all midwives exerted, or were educated in before start of the project. The correlations between GWG and the specific activities (pedometers, walking poles, aqua aerobics) were non-significant, which is in line with previous findings that extra activities do not always have the expected effect [28, 35–38]. The women in the MM intervention described the opportunity to set their own goals for lifestyle change as crucial, and experienced as supportive being in a group setting with other obese pregnant women [42].

An important result of the present study is that the midwives had the opportunity to develop skills for working with obesity and lifestyle issues in the everyday clinic, a topic that midwives in earlier research had expressed having difficulties with [43, 44]. The midwives thus had the opportunity of being empowered to see that their advice would make a difference, since feeling confident in giving advice on GWG is an important predictor of higher guideline adherence [45]. To feel confident and be able to accomplish an efficient and worthy handling of obesity, midwives should have access to nutrition and lifestyle expertise [4].

A strength of the MM programme is that it was population-based and that the women who were eligible for MM were from geographically as well as socio-economically similar compositions. Women with languages other than Swedish were also invited, since it was possible to use interpreters. To avoid biased results caused by an over-representation of highly motivated women, the intervention was delivered through the standard antenatal care system. MM was originally designed as a development project, and a further strength is that the midwives were not involved in the project because they had a particular interest, but were representative of the regular staff. Another strength is that the weight of the woman in the beginning of pregnancy was registered, not reported by the woman, as is often the case in similar studies.

A limitation is that the intervention was not randomised. Also, the area first selected for the control group did not recruit enough women, which led to extending to an adjacent area. However, all three areas were expected to have similar sociodemographic structures. Analyses were adjusted for socioeconomic differences on an individual level.

Another limitation is that even though the MM project was intended to reach all women with BMI ≥30 entering pregnancy, it turned out that 35% were not invited. The low contact level might have been due to midwives neglecting or forgetting to inform women, or abstaining because of a full agenda. The fact that not all midwives and staff feel comfortable in addressing women with obesity has been described elsewhere [43, 46, 47], and may explain why only 65% of the women were asked about participation. Correspondingly, the explanation for why only 62% of the women who were approached chose to participate could be that more negative attitudes towards being pregnant have been reported by women with obesity [48], as well as more unpleasant experiences from attending health care services [43, 46].

The fact that 38% of women declined participation might be explained by their not wanting or feeling able to adhere to the intervention, or being less health literate [49]. A possible selection bias is that the most motivated women opted to join [50]. Both the midwives who invited the women and the women accepting participation (as interventions or controls) may have been more comfortable in dealing with lifestyle issues (the midwife) [45, 46] and had a higher readiness to cope with lifestyle changes (the woman) [50]. Since less than one third of the women in the intervention group fulfilled the criterion of adherence to the study protocol, the conclusions of the PP population are drawn from a rather small proportion of those eligible for participation.

On the other hand, participation in lifestyle interventions in pregnancy is reported to be low, with 40–60% of women eligible to participate declining to do so [44]. A reason for the relatively high participation rate in the Mighty Mums programme could be the possibility of exercising one’s own choice regarding which areas to focus on or which activities to take part in. This in turn lowered the numbers of women participating in the separate activities, and individuals may have missed out on certain aspects of the intervention. Attracting the women to participate is thus of paramount importance, and the person-centred approach with individualised advice formed the base of Mighty Mums.

A related possible source of bias is that the women taking part in the intervention to a greater extent were born in countries other than Sweden, had higher use of interpreters, and were more often not engaged in work. Also, more women in the intervention than in the control group were in Obese Class III (BMI ≥40) and fewer were in the lowest Obese Class I (BMI 30.0–34.9). Higher BMI may have contributed to a lower GWG in the intervention group compared to controls, since GWG usually is lower in women with higher BMI [2, 19]. The challenge of counselling women with obesity and eating disorders has been described by midwives [51], and pregnant women with obesity have asked for culturally adapted programmes [52]. Being born in another country and being less fluent in Swedish may have negatively affected the ability to keep GWG below the determined limit, due to difficulties in understanding and assimilating the information and advice from the midwife. On the other hand, midwives in areas with higher socio-economic and cultural demands might have had to develop certain working skills to cope with this, since counselling women from other cultures is described as a certain challenge [51]. However, the results from this study indicate that the intervention was as relevant to women with a foreign background as to those born in Sweden, potentially due to its person-centred focus on the women’s own capabilities.

Women in the control group may have been influenced by the ongoing MM project, since there is formal and informal communication between midwives, and pregnant women move between areas and voluntarily tell each other pregnancy-related health tips. Women in the control group may also have been referred to a dietician or physiotherapist, taken part in community activities related to lifestyle or other issues independent of the project, or enrolled in other health-related research studies. These circumstances could in reality have decreased the differences between women in the intervention and control groups.

Another limitation is that the intervention programme with free choice of activities makes it difficult to differentiate exactly which parts of the MM intervention contributed to the difference in GWG between the intervention and control groups. The variety in support and activities and the possibility to choose may be factors contributing to success, but it is difficult to define which measure was most effective within the current study design. The extra time with the midwife or contact with the dietician, both weakly correlating with GWG, may also be of importance. Being weighed at every visit has been described with conflicting results [26, 27], and it is unclear whether this contributed to limiting weight gain. However, women in the MM intervention reported that being weighed regularly encouraged them to continue the positive lifestyle changes [42]. Another factor influencing GWG could be the network that was formed with the surrounding community and health centres.

Two extra appointments with the midwife were planned for the intervention group. The extra time with the midwife, as such, and not the content of the intervention visits, may have helped empower the women in the intervention to succeed with the lifestyle project. In the pilot study of MM, where visits to the midwife were counted manually, there was a similar number of visits among women in the intervention and women in the control group [29]. In the full study, however, it was not possible to obtain reliable data on the number of midwife visits for all women, due to differences in routines for reporting to the register, both in time and between areas.

Also, there are concerns about how well the effect of an intervention like MM can be studied, since pregnant women choosing to enter a lifestyle intervention will have a high motivation to make healthy changes during pregnancy, regardless of being in a study or not [37].

The low participation in the MM intervention might be surprising, since pregnancy, preconception, and postnatal periods often are viewed as important and timely stages in the life course for public health intervention [53]. Also, for the pregnant woman with obesity, the health-promoting ambition of the health care service can result in additional demands. It is likely that this is not the first time the woman is addressing concerns about her body weight. The woman’s acceptance of her actual weight and lack of motivation for lifestyle change, as well as sensitivity to being scrutinised and observed for weight matters, has been suggested to negatively impact the possibility of succeeding in restricting GWG and may have hindered some women from participating [48, 54]. The fact that the public health and community services generally lack structured maternal obesity objectives aggravates the possibility of succeeding with lifestyle interventions and calls for more strategic and national support concerning evidence and guidance to plan, develop, and implement effective maternal obesity services [47].

The many barriers that exist for both women and health care providers affect the successful initiation of behavioural change during pregnancy [44]. Midwives describe pregnancy as an ideal time for interventions concerning health among pregnant women, and say that they require support and better cooperation with other healthcare professionals to be able to carry forward greater collaboration with the women they care for [55]. Person-centred care in pregnancy is sparsely studied, and the extent to which person-centred care may improve health outcomes and satisfaction with care in this population needs further research [56].

## Conclusions

This study, which is based on relatively modest changes in the routine visits in primary care, shows that it is possible to guide the pregnant woman with obesity towards everyday lifestyle changes that decrease GWG and lessen weight retention after pregnancy. The number of visits with logbook activity on both food and physical activity as well as dietician consultation correlated significantly with GWG. The individual choice of level of activity and engagement, as well as the personal support and documenting in the logbook, may also be factors in success. However, measures need to be evaluated to have a larger proportion of participants taking full advantage of the programme, and future studies are warranted to put strategies in antenatal care into perspective regarding the whole health care system and society’s handling of overweight and obesity in pregnant women.

### Implications for clinical practice

The findings in this study suggest that a programme starting in early pregnancy, monitoring weight regularly and with an opportunity to discuss nutrition and physical activity with the midwife or other professionals throughout pregnancy, can be an important part of active antenatal care concerning lifestyle issues. Also, the postpartum check-up may be an opportunity for the woman with obesity to be addressed about her current weight and lifestyle and offered further monitoring in primary care. However, for an optimal effect, women need to receive better information on risks and advice on losing weight even before getting pregnant [57].

Activities in the intervention programme that correlated significantly with GWG (extra midwife visits, advice on food and physical activity, and dietician consultation; Table 4), together with mandatory weighing, have been picked up in regional guidelines for antenatal care. However, the implementation of guidelines and optimal antenatal care of obesity require a supportive management and a general consensus in the health care organisation that obesity and overweight are important issues. Further involvement with person-centred care may enhance the outcome of similar interventions in the future.

## Abbreviations

BMI: Body mass index
GWG: Gestational weight gain
IOM: Institute of Medicine
MI: Motivational interviewing
MM: Mighty Mums

## References

[1] Zeitlin J, Mohangoo A, Delnord M (2010). European perinatal health report health and care of pregnant women and babies in Europe in 2010.

[2] Pregnancy register, yearly report 2016. Stockholm: Quality Register Center; 2016.

[3] Holowko N, Chaparro MP, Nilsson K, Ivarsson A, Mishra G, Koupil I, Goodman A (2015). Social inequality in pre-pregnancy BMI and gestational weight gain in the first and second pregnancy among women in Sweden. J Epidemiol Community Health.

[4] Campbell EE, Dworatzek PD, Penava D, de Vrijer B, Gilliland J, Matthews JI, Seabrook JA (2016). Factors that influence excessive gestational weight gain: moving beyond assessment and counselling. J Matern Fetal Neonatal Med.

[5] Drake AJ, Reynolds RM (2010). Impact of maternal obesity on offspring obesity and cardiometabolic disease risk. Reproduction.

[6] Stamnes Koepp UM, Frost Andersen L, Dahl-Joergensen K, Stigum H, Nass O, Nystad W (2012). Maternal pre-pregnant body mass index, maternal weight change and offspring birthweight. Acta Obstet Gynecol Scand.

[7] Marchi J, Berg M, Dencker A, Olander EK, Begley C (2015). Risks associated with obesity in pregnancy, for the mother and baby: a systematic review of reviews. Obes Rev.

[8] Begum F, Colman I, McCargar LJ, Bell RC (2012). Gestational weight gain and early postpartum weight retention in a prospective cohort of Alberta women. J Obstet Gynaecol Can.

[9] Fraser A, Tilling K, Macdonald-Wallis C, Hughes R, Sattar N, Nelson SM, Lawlor DA (2011). Associations of gestational weight gain with maternal body mass index, waist circumference, and blood pressure measured 16 y after pregnancy: the Avon Longitudinal Study of Parents and Children (ALSPAC). Am J Clin Nutr.

[10] Goldstein R, Teede H, Thangaratinam S, Boyle J (2016). Excess gestational weight gain in pregnancy and the role of lifestyle intervention. Semin Reprod Med.

[11] Ludwig DS, Currie J (2010). The association between pregnancy weight gain and birthweight: a within-family comparison. Lancet.

[12] Tie HT, Xia YY, Zeng YS, Zhang Y, Dai CL, Guo JJ, Zhao Y (2014). Risk of childhood overweight or obesity associated with excessive weight gain during pregnancy: a meta-analysis. Arch Gynecol Obstet.

[13] Sridhar SB, Darbinian J, Ehrlich SF, Markman MA, Gunderson EP, Ferrara A, Hedderson MM (2014). Maternal gestational weight gain and offspring risk for childhood overweight or obesity. Am J Obstet Gynecol.

[14] Leonard SA, Petito LC, Rehkopf DH, Ritchie LD, Abrams B (2017). Weight gain in pregnancy and child weight status from birth to adulthood in the United States. Pediatr Obes.

[15] Amorim AR, Rossner S, Neovius M, Lourenco PM, Linne Y (2007). Does excess pregnancy weight gain constitute a major risk for increasing long-term BMI?. Obesity (Silver Spring).

[16] Walter JR, Perng W, Kleinman KP, Rifas-Shiman SL, Rich-Edwards JW, Oken E (2015). Associations of trimester-specific gestational weight gain with maternal adiposity and systolic blood pressure at 3 and 7 years postpartum. Am J Obstet Gynecol.

[17] IOM American Institute of Medicine (2009). Weight gain during pregnancy: reexamining the guidelines.

[18] Cedergren MI (2007). Optimal gestational weight gain for body mass index categories. Obstet Gynecol.

[19] Blomberg M (2011). Maternal and neonatal outcomes among obese women with weight gain below the new Institute of Medicine recommendations. Obstet Gynecol.

[20] Muktabhant B, Lawrie TA, Lumbiganon P, Laopaiboon M (2015). Diet or exercise, or both, for preventing excessive weight gain in pregnancy. Cochrane Database Syst Rev.

[21] International Weight Management in Pregnancy (i-WIP) Collaborative Group (2017). Effect of diet and physical activity based interventions in pregnancy on gestational weight gain and pregnancy outcomes: meta-analysis of individual participant data from randomised trials. BMJ.

[22] Thangaratinam S, Rogozinska E, Jolly K, Glinkowski S, Roseboom T, Tomlinson JW, Kunz R, Mol BW, Coomarasamy A, Khan KS (2012). Effects of interventions in pregnancy on maternal weight and obstetric outcomes: meta-analysis of randomised evidence. BMJ.

[23] Agha M, Agha RA, Sandall J (2014). Interventions to reduce and prevent obesity in pre-conceptual and pregnant women: a systematic review and meta-analysis. PLoS One.

[24] Gardner B, Wardle J, Poston L, Croker H (2011). Changing diet and physical activity to reduce gestational weight gain: a meta-analysis. Obes Rev.

[25] Zhang S, Rattanatray L, Morrison JL, Nicholas LM, Lie S, McMillen IC (2011). Maternal obesity and the early origins of childhood obesity: weighing up the benefits and costs of maternal weight loss in the periconceptional period for the offspring. Exp Diabetes Res.

[26] Jeffries K, Shub A, Walker SP, Hiscock R, Permezel M (2009). Reducing excessive weight gain in pregnancy: a randomised controlled trial. Med J Aust.

[27] Brownfoot FC, Davey MA, Kornman L (2016). Routine weighing to reduce excessive antenatal weight gain: a randomised controlled trial. BJOG.

[28] Claesson IM, Sydsjo G, Brynhildsen J, Cedergren M, Jeppsson A, Nystrom F, Sydsjo A, Josefsson A (2008). Weight gain restriction for obese pregnant women: a case-control intervention study. BJOG.

[29] Haby K, Glantz A, Hanas R, Premberg A (2015). Mighty Mums - an antenatal health care intervention can reduce gestational weight gain in women with obesity. Midwifery.

[30] Jacobs G (2011). “Take control or lean back?” barriers to practicing empowerment in health promotion. Health Promot Pract.

[31] Rollnick S, Butler CC, Kinnersley P, Gregory J, Mash B (2010). Motivational interviewing. BMJ.

[32] McCormack B, McCance TV (2006). Development of a framework for person-centred nursing. J Adv Nurs.

[33] Johansson K, Hutcheon JA, Stephansson O, Cnattingius S (2016). Pregnancy weight gain by gestational age and BMI in Sweden: a population-based cohort study. Am J Clin Nutr.

[34] Wolff S, Legarth J, Vangsgaard K, Toubro S, Astrup A (2008). A randomized trial of the effects of dietary counseling on gestational weight gain and glucose metabolism in obese pregnant women. Int J Obes.

[35] Shirazian T, Monteith S, Friedman F, Rebarber A (2010). Lifestyle modification program decreases pregnancy weight gain in obese women. Am J Perinatol.

[36] Vinter CA, Jensen DM, Ovesen P, Beck-Nielsen H, Jorgensen JS (2011). The LiP (Lifestyle in Pregnancy) study: a randomized controlled trial of lifestyle intervention in 360 obese pregnant women. Diabetes Care.

[37] Bogaerts AF, Devlieger R, Nuyts E, Witters I, Gyselaers W, Van den Bergh BR. Effects of lifestyle intervention in obese pregnant women on gestational weight gain and mental health: a randomized controlled trial. Int J Obes (Lond). 2013;37(6):814–21. 10.1038/ijo.2012.162 23032404

[38] Quinlivan JA, Lam LT, Fisher J (2011). A randomised trial of a four-step multidisciplinary approach to the antenatal care of obese pregnant women. Aust N Z J Obstet Gynaecol.

[39] Dencker A, Premberg A, Olander EK, McCourt C, Haby K, Dencker S, Glantz A, Berg M (2016). Adopting a healthy lifestyle when pregnant and obese - an interview study three years after childbirth. BMC Pregnancy Childbirth.

[40] Tanentsapf I, Heitmann BL, Adegboye AR (2011). Systematic review of clinical trials on dietary interventions to prevent excessive weight gain during pregnancy among normal weight, overweight and obese women. BMC Pregnancy Childbirth.

[41] Oteng-Ntim E, Varma R, Croker H, Poston L, Doyle P (2012). Lifestyle interventions for overweight and obese pregnant women to improve pregnancy outcome: systematic review and meta-analysis. BMC Med.

[42] Fieril DP, Olsen PF, Glantz D, Premberg DA (2017). Experiences of a lifestyle intervention in obese pregnant women - a qualitative study. Midwifery.

[43] Furness PJ, McSeveny K, Arden MA, Garland C, Dearden AM, Soltani H (2011). Maternal obesity support services: a qualitative study of the perspectives of women and midwives. BMC Pregnancy Childbirth.

[44] Dodd JM, Briley AL (2017). Managing obesity in pregnancy - an obstetric and midwifery perspective. Midwifery.

[45] Herring SJ, Platek DN, Elliott P, Riley LE, Stuebe AM, Oken E (2010). Addressing obesity in pregnancy: what do obstetric providers recommend?. J Women's Health (Larchmt).

[46] Heslehurst N, Moore H, Rankin J, Ells LJ, Wilkinson JR, Summberbell CD (2011). How can maternity services be developed to effectively address maternal obesity? A qualitative study. Midwifery.

[47] Smith SA, Heslehurst N, Ells LJ, Wilkinson JR (2011). Community-based service provision for the prevention and management of maternal obesity in the North East of England: a qualitative study. Public Health.

[48] Nyman VM, Prebensen AK, Flensner GE (2010). Obese women's experiences of encounters with midwives and physicians during pregnancy and childbirth. Midwifery.

[49] Kennen EM, Davis TC, Huang J, Yu H, Carden D, Bass R, Arnold C (2005). Tipping the scales: the effect of literacy on obese patients’ knowledge and readiness to lose weight. South Med J.

[50] Tanvig M (2014). Offspring body size and metabolic profile - effects of lifestyle intervention in obese pregnant women. Dan Med J.

[51] Wennberg A (2015). Pregnant women and midwives are not in tune with each other about dietary counselling - studies in Swedish antenatal care. Umeå University.

[52] Mills A, Schmied VA, Dahlen HG (2013). Get alongside us’, women’s experiences of being overweight and pregnant in Sydney, Australia. Matern Child Nutr.

[53] Phelan S (2010). Pregnancy: a “teachable moment” for weight control and obesity prevention. Am J Obstet Gynecol.

[54] Phelan S, Phipps MG, Abrams B, Darroch F, Schaffner A, Wing RR (2011). Practitioner advice and gestational weight gain. J Women's Health (Larchmt).

[55] Aquino MR, Edge D, Smith DM (2015). Pregnancy as an ideal time for intervention to address the complex needs of black and minority ethnic women: views of British midwives. Midwifery.

[56] Olander EK, Berg M, McCourt C, Carlstrom E, Dencker A (2015). Person-centred care in interventions to limit weight gain in pregnant women with obesity - a systematic review. BMC Pregnancy Childbirth.

[57] Forsum E, Brantsaeter AL, Olafsdottir AS, Olsen SF, Thorsdottir I. Weight loss before conception: a systematic literature review. Food Nutr Res. 2013;57. 10.3402/fnr.v57i0.20522. Epub 2013 Mar 13. 10.3402/fnr.v57i0.20522 PMC3597776 23503117

